# Care and control under dual transformation pressures: a three-wave multi-source study of paternalistic leadership, ambivalent psychological safety, surface acting, and employee voice in Malaysia’s service industry

**DOI:** 10.3389/fpsyg.2026.1923937

**Published:** 2026-10-14

**Authors:** Yuanyuan Ping, Sharmila A. P. Jayasingam, Raida Binti Abu Bakar

**Affiliations:** Faculty of Business and Economics, University of Malaya, Kuala Lumpur, Malaysia

**Keywords:** ambivalent psychological safety, authoritarianism, benevolence, employee voice, paternalistic leadership, prohibitive voice, promotive voice, surface acting

## Abstract

Malaysia’s service industry is undergoing simultaneous artificial-intelligence transformation and statutory minimum-wage pressure, creating a context in which service leaders are expected to show individualized care while also enforcing stronger productivity discipline. Drawing on conservation of resources theory and employee voice research, this three-wave, multi-source study examined whether the benevolent-authoritarian duality of paternalistic leadership was associated with promotive and prohibitive voice through ambivalent psychological safety and emotional resource depletion, and whether surface acting conditioned this pattern. The analytic sample comprised 360 service employee-supervisor dyads nested within 72 supervisors and 18 organizations/branches. Employees reported paternalistic leadership and surface acting at Time 1 and ambivalent psychological safety and emotional resource depletion at Time 2; supervisors rated promotive and prohibitive voice at Time 3. The benevolence x authoritarianism interaction was positively associated with ambivalent psychological safety (b = 0.400, *p* < 0.001), which was positively associated with emotional resource depletion (b = 0.390, *p* < 0.001). Surface acting strengthened the ambivalent-safety-to-depletion association (b = 0.151, *p* = 0.001). Resource depletion was negatively associated with promotive voice (b = −0.442, *p* < 0.001) and prohibitive voice (b = −0.247, *p* < 0.001), with a stronger association for promotive voice. Conditional indirect associations were stronger under high surface acting. These observational findings are consistent with the proposition that care-control leadership can reassure employees while being associated with lower emotional resources for constructive voice in AI- and wage-pressure service contexts.

## 1 Introduction

Employee voice is critical for service organizations because frontline employees often notice customer friction, process failures, and improvement opportunities before formal reporting systems do. Voice is particularly important during digital service transformation, when new service scripts, AI-assisted workflows, and technology-enabled monitoring can create operational problems that managers cannot easily observe from a distance (Morrison, 2011, 2014; Maynes and Podsakoff, 2014).

In Malaysia’s service industry, AI transformation is occurring alongside wage-related productivity pressure. These dual pressures make employee suggestions and warnings valuable, but they can also make supervisors more controlling and employees more cautious. A suggestion about an AI-supported workflow may be interpreted as learning-oriented input, whereas the same suggestion may also be interpreted as resistance to mandated change.

Voice research distinguishes promotive voice, which offers ideas for improving future functioning, from prohibitive voice, which raises concerns about harmful practices or risks (Van Dyne and LePine, 1998; Liang et al., 2012; Chamberlin et al., 2017). Both forms involve interpersonal risk, but they differ in resource demand: promotive voice requires constructive idea generation, whereas prohibitive voice is often anchored in a visible problem.

Leadership cues shape whether employees are willing to take these risks. Prior work shows that leader openness and psychological safety can encourage voice (Kahn, 1990; Edmondson, 1999; Detert and Burris, 2007; Burris, 2012), yet many service employees do not experience leadership as uniformly open or uniformly threatening. Supervisors may provide personal care while also emphasizing hierarchy, discipline, and productivity.

Paternalistic leadership captures this care-control tension. Benevolence communicates individualized concern and protection, whereas authoritarianism communicates hierarchy, obedience, and leader authority (Farh and Cheng, 2000; Cheng et al., 2004; Pellegrini and Scandura, 2008; Zhang et al., 2015). The present study focuses on the dual presence of benevolence and authoritarianism because this configuration is likely under AI- and wage-pressure service conditions.

The core mechanism proposed here is ambivalent psychological safety, defined as the simultaneous activation of interpersonal protection and interpersonal caution regarding the same focal act of speaking up. Conventional psychological safety concerns the perceived consequences of interpersonal risk taking (Edmondson and Lei, 2014), whereas low psychological safety denotes a generally unfavorable assessment of those consequences. Fear of speaking up is likewise predominantly aversive. Ambivalent psychological safety is different: the employee concurrently recognizes a positive cue (the leader cares and may protect the relationship) and a negative cue (the leader values obedience and may sanction challenge). Because neither evaluation cancels the other, the employee remains both reassured and vigilant. This co-activation, rather than a midpoint on a single safety continuum, is the construct examined here (Ashforth et al., 2014).

Surface acting further intensifies this process. Service employees often display required emotions that differ from what they actually feel, and this emotional labor can drain self-regulatory resources (Ashforth and Humphrey, 1993; Grandey, 2000; Brotheridge and Lee, 2003; Hülsheger and Schewe, 2011). Under high surface acting, employees may have fewer resources left to process mixed leadership cues and prepare voice behavior.

This study contributes to the literature by testing a care-control-resource model in which benevolent and authoritarian paternalistic leadership jointly predict ambivalent psychological safety, ambivalent safety predicts emotional resource depletion, surface acting strengthens this depletion process, and depletion differentially reduces promotive and prohibitive voice. The study also contributes methodologically by combining measurement-model evidence with composite-score path analysis and latent SEM robustness checks.

## 2 Theory and hypotheses

The theoretical model treats benevolent and authoritarian paternalistic leadership as a configuration rather than as two isolated predictors. Benevolence supplies a relationally positive cue by signaling support and protection; authoritarianism supplies a simultaneously restrictive cue by signaling hierarchy, obedience, and possible sanction for disagreement. Organizational ambivalence arises when positive and negative orientations toward the same target coexist (Ashforth et al., 2014). Accordingly, the focal state is not produced by care or control alone, but by employees’ need to hold and reconcile both evaluations when deciding whether the same supervisor is safe to challenge.

As shown in Figure 1, the model links the benevolence-authoritarianism configuration to ambivalent psychological safety, emotional resource depletion, and two forms of employee voice. Surface acting is positioned as a resource-intensifying boundary condition.

**Figure 1:**
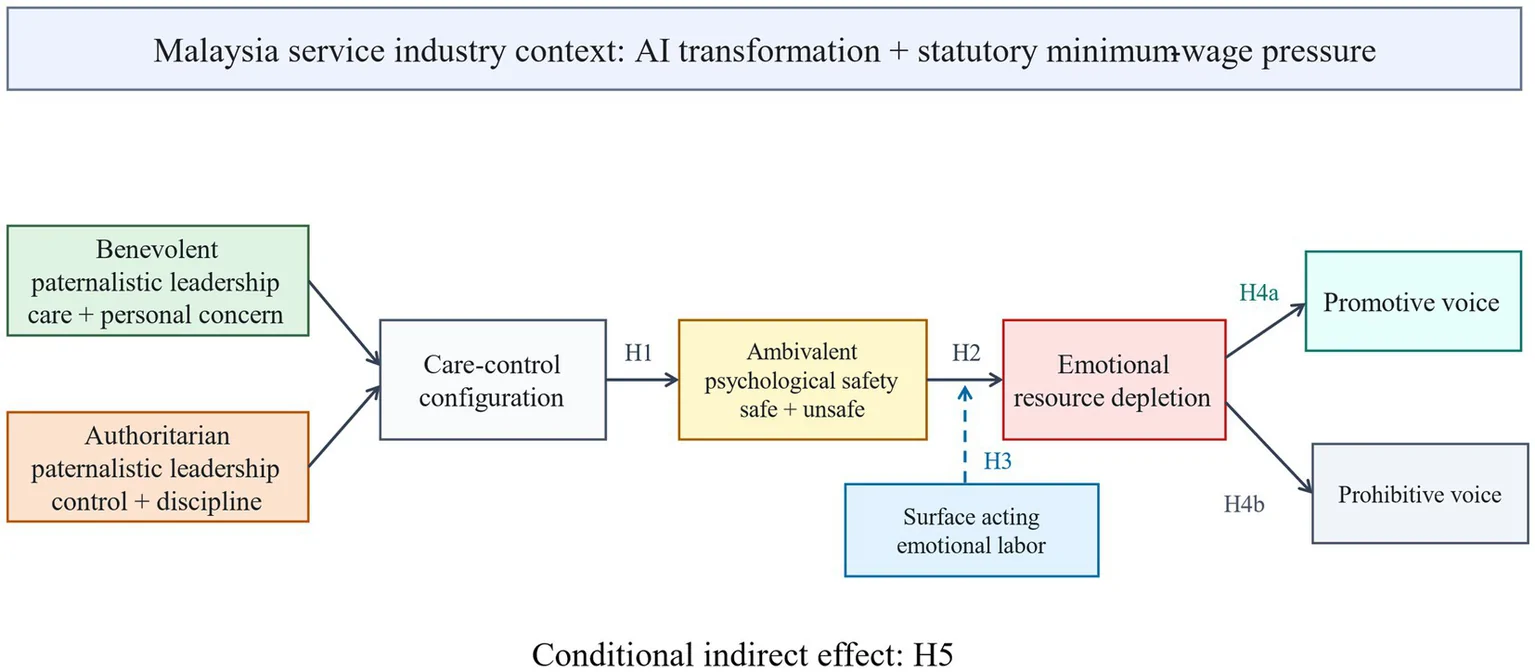
Conceptual model of paternalistic leadership, ambivalent psychological safety, surface acting, resource depletion, and employee voice.

As shown in Figure 2, the high-benevolence/high-authoritarianism condition is expected to be the most psychologically ambivalent. In this condition, employees may feel personally protected but professionally constrained, making it difficult to judge whether voice will be welcomed or sanctioned.

**Figure 2:**
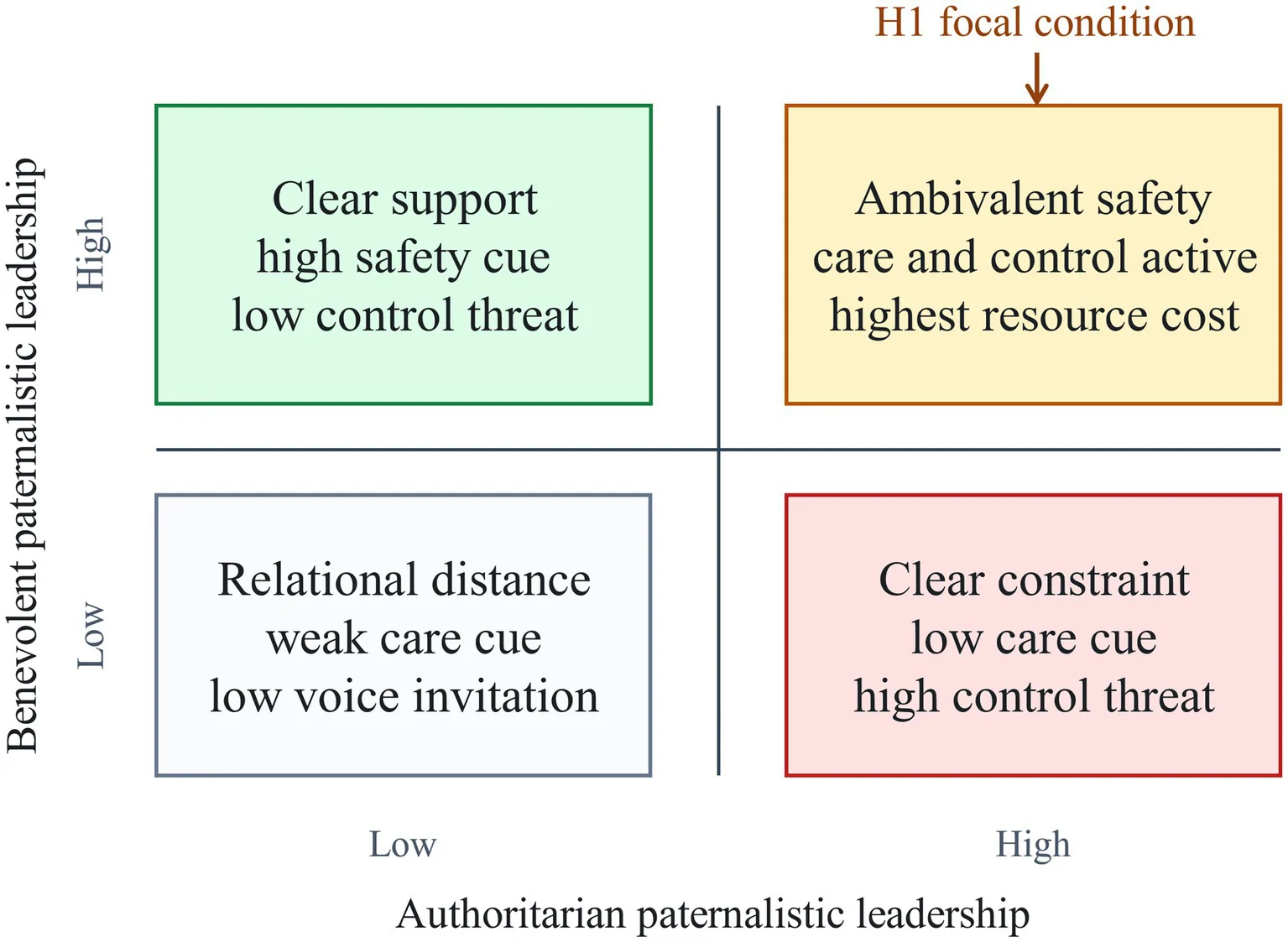
Benevolence-authoritarianism configurations and the focal ambivalent-safety condition.

*H1*: Benevolent and authoritarian paternalistic leadership will interact in predicting ambivalent psychological safety, such that ambivalent psychological safety will be highest when both benevolence and authoritarianism are high.

Ambivalent psychological safety should be positively associated with resource depletion because mixed cues prevent a simple approach-or-avoid decision. Before speaking, employees must repeatedly scan the leader’s behavior, infer which cue will govern the current interaction, rehearse wording that preserves respect, and inhibit responses that might be interpreted as disobedience. Conservation of resources theory suggests that this sustained monitoring and self-regulation draw on emotional energy (Hobfoll, 1989, 2001). The argument therefore concerns the resource cost of unresolved co-activation, not the effect of merely having a low safety score.

*H2*: Ambivalent psychological safety will be positively related to emotional resource depletion.

Surface acting should strengthen this resource-loss process through resource competition. Surface acting requires employees to monitor felt emotion, suppress or mask discrepant affect, and produce a role-required display (Grandey, 2000; Brotheridge and Lee, 2003). Ambivalent leader cues simultaneously require monitoring, interpretation, and response inhibition. When both demands occur, the same limited self-regulatory pool must support customer-facing display control and leader-focused sensemaking. Employees high in surface acting therefore have less spare capacity to resolve whether care or control will govern a voice episode, making a given level of ambivalent psychological safety more strongly associated with emotional resource depletion. This mechanism makes surface acting a theoretically proximal boundary condition rather than a generic employee characteristic.

*H3*: Surface acting will strengthen the positive relationship between ambivalent psychological safety and emotional resource depletion.

Resource depletion should reduce employee voice because speaking up requires attention, confidence, and discretionary effort. The effect should be stronger for promotive voice because improvement suggestions require future-oriented idea generation, whereas prohibitive voice may be sustained when a visible risk makes warning behavior urgent.

*H4a*: Emotional resource depletion will be negatively related to promotive voice.

*H4b*: Emotional resource depletion will be negatively related to prohibitive voice, but this relationship will be weaker than the relationship between emotional resource depletion and promotive voice.

Together, these arguments imply a moderated serial indirect process. Following moderated mediation logic (Preacher et al., 2007), the indirect relationship from the care-control configuration to voice should be stronger when surface acting is high and should be more negative for promotive than prohibitive voice.

*H5*: The indirect relationship between the high-benevolence/high-authoritarianism configuration and employee voice through ambivalent psychological safety and emotional resource depletion will be stronger when surface acting is high, and the conditional indirect effect will be more negative for promotive voice than for prohibitive voice.

## 3 Materials and methods

### 3.1 Research design and participants

This study used a three-wave, multi-source field-survey structure in Malaysia’s service industry. Fieldwork was organized from 6 May to 29 July 2025 through Qualtrics survey links distributed by HR liaisons, branch managers, team leaders, email invitations, WhatsApp groups, and QR-code briefings. The analytic dataset contained full-time and part-time service employees who had direct interaction with customers, digital service systems, or AI-supported service workflows, together with their immediate supervisors. Service subsectors included hospitality, retail, food and beverage, banking services, call centers, logistics, and platform-mediated services.

The design was selected to match the temporal logic of the model. Leadership and surface acting were measured first because they represent the contextual and emotional-labor conditions employees bring into later psychological processing. Ambivalent psychological safety and resource depletion were measured at the second wave because they represent intervening states that should emerge after employees experience leadership cues and emotional display demands. Voice was rated by supervisors at the third wave because the outcomes are enacted behaviors that supervisors are positioned to observe.

Organizational access was arranged through designated HR or branch-level liaisons. Each participating site received a coded identifier (MY-SVC-01 to MY-SVC-18), and the analytic file retained only anonymized organization codes, service subsector, region, size band, recruitment channel, and survey platform. Legal company names were not reported because the participating organizations allowed access on a confidentiality basis.

Participants were eligible when they worked in a service role involving direct customer interaction, digital service systems, AI-supported workflows, or service-performance monitoring. This inclusion logic ensured that respondents were exposed to the dual transformation pressures relevant to the study. Supervisors provided outcome ratings only for employees with whom they had regular work contact, which strengthened the interpretability of the voice ratings.

The three-wave design separated predictors, mechanisms, and outcomes. At Time 1 (6–24 May 2025), employees evaluated paternalistic leadership and surface acting. At Time 2 (10–28 June 2025), employees reported ambivalent psychological safety and emotional resource depletion. At Time 3 (15–29 July 2025), supervisors rated employees’ promotive and prohibitive voice. This temporal and source separation reduced same-source inflation and aligned the analytic structure with the proposed resource-loss process.

The three waves were matched using confidential identification codes rather than names in the analytic file. This procedure allowed responses to be linked across time while reducing participants’ concern that individual answers would be disclosed to supervisors. Employees completed the Time 1 and Time 2 measures, whereas supervisors completed the Time 3 voice ratings. This source separation is important because the model includes both employee psychological states and supervisor-observed voice outcomes.

The multi-source design also reduces common method concerns. Although no survey design can fully eliminate such concerns, separating predictors, mechanisms, and outcomes across time and source follows standard recommendations for reducing artifactual covariance (Podsakoff et al., 2003, 2012). This is particularly important for leadership and voice research because employees’ affective states can otherwise color both predictor and outcome ratings.

The final analytic sample included 360 employee-supervisor dyads nested within 72 supervisors and 18 anonymized participating organizations/branches coded MY-SVC-01 to MY-SVC-18. The participating sites were located in Klang Valley, Selangor, Penang, Johor, and Sabah; legal company names were not reported to preserve organizational confidentiality. Employees were 57.8% female and 42.2% male. The average age was 31.25 years (SD = 7.47), and average organizational tenure was 39.12 months (SD = 26.62). Education levels were bachelor (145), diploma (127), secondary (68), and postgraduate (20). The largest service-sector groups were food and beverage (73), hospitality (63), retail (58), banking services (46), platform services (42), call centers (42), and logistics (36).

The analytic estimand is at the employee-dyad level because leadership, surface acting, ambivalent psychological safety, and depletion represent employee-specific perceptions or states, and each supervisor supplied a separate voice rating for each matched employee. Nevertheless, the 360 dyads were nested within 72 supervisors (mean cluster size = 5) and 18 organizations/branches (mean cluster size = 20). A full three-level latent-interaction model was not selected as the primary specification because only 18 level-3 units were available and the focal hypotheses concern employee-level paths. We therefore retained the composite-score model as the primary analysis and re-estimated inference using finite-sample-corrected cluster-robust covariance matrices separately at the supervisor and organization levels (Cameron and Miller, 2015). Organization-cluster bootstrap resampling was additionally used for the conditional indirect effects.

The sector distribution reflects several service settings in which AI transformation and wage pressure are likely to be visible to employees: hospitality, retail, food and beverage, banking services, call centers, logistics, and platform-mediated services. These subsectors differ in customer-contact intensity and digitalization, but they share a common need for employees to notice problems and communicate improvement ideas.

As shown in Figure 3, the design separated predictors, mediators, and outcomes across time and source. As shown in Table 1, the collection plan specified the wave, source, measures, fieldwork window, survey platform, and distribution channel for each stage. This structure supports a stronger test of the proposed process than a single-source cross-sectional design because leadership and surface acting precede the psychological mechanism, and supervisor-rated voice follows the resource-depletion mechanism.

**Figure 3:**
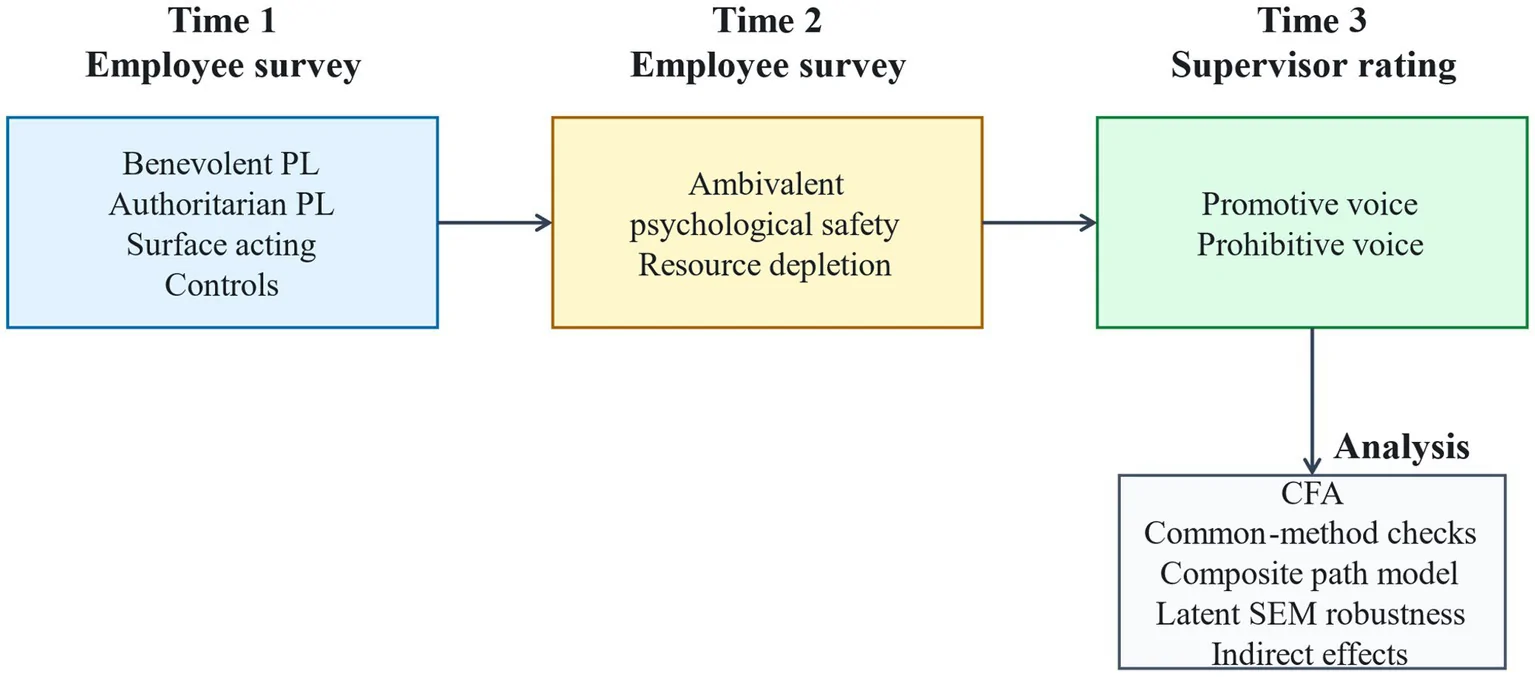
Three-wave, multi-source empirical design for testing the model.

**Table 1:** Data collection design for the original research study.

Wave	Source	Measures	Purpose
Time 1	Employees via qualtrics link	Benevolent PL, authoritarian PL, surface acting, controls	6–24 May 2025; HR/branch-liaison email, WhatsApp, and QR-code distribution.
Time 2	Employees via qualtrics link	Ambivalent psychological safety, emotional resource depletion	10–28 June 2025; matched follow-up link sent through the same organizational liaison.
Time 3	Supervisors via qualtrics rating link	Promotive voice, prohibitive voice	15–29 July 2025; supervisors rated matched employees after the employee waves closed.
Analysis	Research team	CFA, composite-score path analysis, latent SEM robustness, common-method checks, and bootstrap indirect effects	Analyze the anonymized three-wave dyad file after measurement, method-bias, and matching checks were complete.

### 3.2 Measures

As shown in Table 2, all multi-item scales used a five-point response format (1 = strongly disagree, 5 = strongly agree). Scale scores were computed as item means, with higher values representing higher levels of the focal construct. Published measures were adapted to the Malaysian service context through minor wording changes that preserved their original construct content. The source and one illustrative item for each measure are reported below to make the operationalization reproducible.

**Table 2:** Measures, sources, and operationalization.

Construct	Source and timing	Operationalization in this study
Benevolent paternalistic leadership	Employee report, Time 1	Five-item composite capturing individualized care, concern, and support from the immediate supervisor.
Authoritarian paternalistic leadership	Employee report, Time 1	Five-item composite capturing authority, discipline, obedience expectations, and hierarchical control.
Ambivalent psychological safety	Employee report, Time 2	Six-item composite capturing the coexistence of felt protection and caution about challenging the supervisor.
Surface acting	Employee report, Time 1	Four-item composite capturing the display of required emotions that are not genuinely felt.
Emotional resource depletion	Employee report, Time 2	Five-item composite capturing emotional fatigue and reduced available psychological resources.
Promotive/prohibitive voice	Supervisor report, Time 3	Separate five-item composites for improvement-oriented promotive voice and risk-oriented prohibitive voice.

Benevolent and authoritarian paternalistic leadership were each measured with five items adapted from Cheng et al. (2004). Benevolence captured individualized concern, support, and attention to employees’ work and personal circumstances; an illustrative item was, “My supervisor shows concern for my life beyond immediate work tasks.” Authoritarianism captured authority, discipline, obedience expectations, and hierarchical control; an illustrative item was, “My supervisor expects employees to follow his or her instructions completely.” Employees rated their immediate supervisor at Time 1. The two dimensions were scored separately and then interacted because the theoretical argument concerns their joint presence.

Ambivalent psychological safety was measured at Time 2 with six items developed for this study by combining the interpersonal-risk focus of psychological safety (Edmondson, 1999; Edmondson and Lei, 2014) with the same-target co-activation criterion in organizational ambivalence theory (Ashforth et al., 2014). Every item referred to the same immediate supervisor and the same domain of speaking-up risk. An illustrative item was, “My supervisor cares about me, yet I am uncertain whether it is safe to disagree with him or her.” Thus, the scale captures simultaneous protection and caution rather than a uniformly unfavorable safety judgment or a primarily fear-based response. Its internal consistency and factorial distinctiveness provide initial measurement evidence; broader convergent, discriminant, and incremental evidence remains a priority for future research.

Surface acting was measured at Time 1 with four items adapted from Brotheridge and Lee (2003). An illustrative item was, “I pretend to have emotions that I do not really feel while serving customers.” Emotional resource depletion was measured at Time 2 with five items adapted from the emotional-exhaustion component of Maslach and Jackson (1981) and framed as currently available emotional energy; an illustrative item was, “I feel emotionally drained by my work.” The wording retained the experienced depletion content while matching the proposed conservation-of-resources mechanism.

Promotive and prohibitive voice were measured at Time 3 with separate five-item supervisor ratings adapted from Liang et al. (2012). An illustrative promotive item was, “This employee proactively suggests ways to improve work procedures.” An illustrative prohibitive item was, “This employee speaks up about practices that could harm the work unit.” Using parallel supervisor-rated measures reduced same-source overlap and preserved the behavioral distinction between improvement-oriented suggestions and risk-oriented warnings.

The measurement strategy therefore mirrored the conceptual model. Leadership dimensions were employee-rated because employees’ interpretation of care and control is the psychological input to ambivalent safety. Psychological mechanisms were employee-rated because ambivalent safety and depletion are internal states. Voice outcomes were supervisor-rated because supervisors observe whether employees actually express suggestions and concerns in the work setting.

Control variables included employee age, gender, education, organizational tenure, supervisor tenure, service subsector, customer-contact intensity, AI exposure, and perceived wage pressure. The main analyses focused on standardized focal variables to keep interpretation transparent.

Controls were selected to account for alternative explanations related to demographic experience, service context, and transformation pressure. Age and organizational tenure may shape confidence and familiarity with speaking up. Education may influence employees’ comfort with process suggestions. Customer-contact intensity, AI exposure, and perceived wage pressure are particularly relevant because they capture the operational conditions that make emotional labor and voice demands more salient.

### 3.3 Analytical strategy

The analysis followed a prespecified sequence (Anderson and Gerbing, 1988). First, confirmatory factor analysis compared the hypothesized seven-factor structure with theoretically plausible alternatives. Second, standardized composite-score path models tested the focal relations. Benevolence and authoritarianism were standardized before multiplication; ambivalent psychological safety and surface acting were treated in the same way. Third, conditional indirect effects were estimated at −1 SD and +1 SD of surface acting. Fourth, a latent-product SEM using double-mean-centered product indicators assessed whether the substantive pattern depended on composite scoring (Marsh et al., 2004; Little et al., 2006). Fifth, every focal composite-score equation was re-estimated with CR1 finite-sample-corrected covariance matrices clustered separately by supervisor (72 clusters, *df* = 71) and organization (18 clusters, *df* = 17). Sixth, H4a and H4b were compared in a stacked-outcome model containing outcome-type interactions with every regressor. Finally, 5,000 organization-cluster bootstrap samples, each resampling entire organizations with all constituent supervisors and employees, were used for the conditional indirect associations. Common method bias was examined with Harman’s first-factor check, a single-factor CFA, and a common latent method-factor CFA.

This design deliberately separates measurement assessment from path estimation. The CFA establishes whether the seven focal constructs are empirically distinguishable; it is not presented as the model that directly generates the hypothesis-test coefficients. The hypothesis tests use standardized composite scores because the theoretical model contains two interactions and conditional indirect effects that are easier to estimate, bootstrap, and interpret in a composite-score path framework when reliabilities are acceptable.

To prevent this two-stage approach from becoming a methodological weak point, the analyses also included a latent-product SEM robustness check. In that model, the benevolence x authoritarianism and ambivalent psychological safety x surface acting terms were represented using product-indicator logic, and the direction, magnitude, and significance pattern were compared with the composite-score path model. The robustness results produced the same substantive inferences.

Conditional indirect effects were computed as the product of (a) the benevolence-by-authoritarianism path to ambivalent psychological safety, (b) the conditional ambivalent-safety-to-depletion path evaluated at −1 SD and +1 SD of surface acting, and (c) the relevant depletion-to-voice path. The original estimates used 1,000 individual-level resamples; the clustering robustness analysis used 5,000 organization-cluster resamples and percentile 95% confidence intervals. Resampling whole organizations preserves both organization-level dependence and the supervisor-within-organization structure. Effects were estimated separately for promotive and prohibitive voice, and their high-surface-acting contrast was calculated within every bootstrap sample.

For the composite-score configuration test, standardized benevolent and authoritarian leadership scores were multiplied to form the benevolence x authoritarianism term. A coefficient of 0.400 therefore represents the change in the benevolence slope associated with a one-standard-deviation increase in authoritarianism. The ambivalent psychological safety x surface acting coefficient is interpreted analogously: 0.151 is the change in the ambivalent-safety slope associated with a one-standard-deviation increase in surface acting. The latent SEM robustness model used double-mean-centered product indicators. Unstandardized path estimates, standard errors, *p* values, and 95% confidence intervals are reported together so that direction, uncertainty, and magnitude are visible.

The analyses are reported transparently so that the theoretical claims can be connected directly to the statistical evidence: H1 corresponds to the benevolence x authoritarianism interaction, H2 to the ambivalent-safety path, H3 to the ambivalent-safety x surface-acting interaction, H4a and H4b to the two depletion-to-voice paths, and H5 to the moderated indirect pattern.

## 4 Results

The observed associations were consistent with the proposed care-control-resource model. The benevolence-by-authoritarianism configuration was positively associated with ambivalent psychological safety; ambivalent psychological safety was positively associated with emotional resource depletion; surface acting strengthened this association; and depletion showed a stronger negative association with promotive voice than with prohibitive voice. Because the data are observational, the temporal ordering and multi-source design strengthen but do not establish causal inference.

### 4.1 Measurement model

The hypothesized seven-factor measurement model was compared with four alternative or diagnostic models. As shown in Table 3, the seven-factor model provided the strongest substantive performance among the tested models (CFI = 0.948, TLI = 0.941, RMSEA = 0.041, SRMR = 0.046). Combining the two voice factors, combining ambivalent psychological safety with resource depletion, or collapsing all indicators into one factor produced poorer performance. The common latent method-factor model was used as a diagnostic check rather than as the retained theoretical model.

**Table 3:** Measurement model comparison results.

Model	Description	Chi-square	*df*	CFI	TLI	RMSEA	SRMR	Decision
M1	Seven-factor model	861.4	539	0.948	0.941	0.041	0.046	Retained
M2	Combine two voice factors	1047.8	545	0.919	0.911	0.051	0.059	Poorer performance
M3	Combine ambivalent safety and depletion	1172.2	545	0.899	0.889	0.057	0.065	Poorer performance
M4	Single-factor model	2446.9	560	0.696	0.671	0.097	0.128	Poorer performance
M5	Seven-factor model with common latent method factor	829.6	506	0.952	0.944	0.042	0.044	Method-factor check

The measurement results are important because several constructs in the model could otherwise be confused conceptually. Ambivalent psychological safety must be distinguishable from emotional resource depletion; otherwise the model would collapse the psychological interpretation of mixed safety into a general strain measure. Similarly, promotive and prohibitive voice must remain distinct because the theory predicts a stronger depletion effect for promotive voice. As shown in Table 3, the seven-factor model performed better than the alternatives that combined these constructs.

The common-method checks were included because Time 1 leadership and surface acting were collected from the same employee source. Harman’s first unrotated factor accounted for 19.3% of item variance, the single-factor CFA performed poorly, and adding a common latent method factor improved model performance only slightly (Delta CFI = 0.004; maximum standardized loading change = 0.041). These results do not eliminate common-method risk, but they reduce the likelihood that a single method factor explains the seven-construct pattern.

Reliability estimates were acceptable for all focal scales, ranging from alpha = 0.819 for surface_acting to alpha = 0.863 for ambivalent_ps. Common method checks did not indicate a dominant method factor: Harman’s first unrotated factor accounted for 19.3% of item variance, the single-factor CFA performed poorly, and adding a common latent method factor changed CFI by only 0.004 with a maximum standardized loading change of 0.041. These results support treating the focal variables as distinct constructs in the analytic dataset.

The reliability pattern also supports the use of composite scale scores. All alpha coefficients exceeded conventional acceptability thresholds, and the range was narrow enough to indicate that no single construct was measured substantially less reliably than the others. This matters for comparing promotive and prohibitive voice because a weaker coefficient for prohibitive voice should not be attributed to poor measurement reliability.

The latent SEM robustness check addressed the remaining measurement-to-structure concern. The robustness model preserved the seven-factor measurement structure while estimating the same substantive paths with latent factors and product indicators. Because its results matched the composite-score path analysis, the manuscript reports the more interpretable composite estimates in Table 4 and treats the latent SEM as a robustness check rather than a replacement analysis.

**Table 4:** Hypothesis testing results.

Hypothesis	Path or effect	Estimate	SE	*p* value	95% CI	Conclusion
H1	Benevolent PL × authoritarian PL → ambivalent psychological safety	0.400	0.045	<0.001	[0.312, 0.488]	Supported
H2	Ambivalent psychological safety → resource depletion	0.390	0.047	<0.001	[0.298, 0.482]	Supported
H3	Ambivalent safety × surface acting → resource depletion	0.151	0.046	=0.001	[0.061, 0.241]	Supported
H4a	Resource depletion → promotive voice	−0.442	0.050	<0.001	[−0.540, −0.344]	Supported
H4b	Resource depletion → prohibitive voice	−0.247	0.054	<0.001	[−0.353, −0.141]	Supported
H5	High-surface-acting indirect contrast: promotive minus prohibitive	−0.042	0.015	—	[−0.076, −0.018]	Supported

### 4.2 Descriptive statistics and correlations

As shown in Table 5, the descriptive statistics showed meaningful dispersion across constructs rather than a uniform midpoint pattern. Scale means ranged from 2.748 to 3.537 and standard deviations ranged from 0.573 to 0.751. Ambivalent psychological safety correlated positively with resource depletion (*r* = 0.450), and resource depletion correlated negatively with promotive voice (*r* = −0.408) and prohibitive voice (*r* = −0.222). These zero-order patterns are consistent with the proposed resource-loss logic and also foreshadow the expected asymmetry across voice forms.

**Table 5:** Descriptive statistics, reliabilities, and correlations.

Variable	Mean	SD	Reliability	1	2	3	4	5	6	7
1. Benevolent PL	3.537	0.656	0.856	—						
2. Authoritarian PL	2.748	0.751	0.846	−0.074	—					
3. Surface acting	3.307	0.718	0.819	0.155	0.254	—				
4. Ambivalent psychological safety	3.085	0.599	0.863	0.130	0.196	0.198	—			
5. Emotional resource depletion	3.257	0.674	0.836	0.263	0.195	0.282	0.450	—		
6. Promotive voice	3.430	0.573	0.824	0.091	−0.196	−0.207	−0.207	−0.408	—	
7. Prohibitive voice	2.882	0.655	0.837	0.099	−0.145	−0.125	−0.172	−0.222	0.062	—

The correlations also show that benevolent and authoritarian paternalistic leadership are not redundant. Their association was small (*r* = −0.074), supporting the decision to model them as separate dimensions whose interaction captures a configuration. Surface acting correlated positively with authoritarian leadership (*r* = 0.254), ambivalent psychological safety (*r* = 0.198), and resource depletion (*r* = 0.282), which is consistent with the argument that emotional labor is connected to both leadership pressure and resource strain.

Promotive and prohibitive voice were only weakly correlated (*r* = 0.062), further supporting the decision to analyze them separately. This weak association is theoretically meaningful because it indicates that employees who offer improvement suggestions are not necessarily the same employees who raise risk warnings. Treating voice as a single outcome would therefore hide important behavioral differences.

### 4.3 Hypothesis tests

As shown in Table 4, the benevolence x authoritarianism interaction was positively associated with ambivalent psychological safety (b = 0.400, SE = 0.045, *p* < 0.001, 95% CI [0.312, 0.488]), supporting H1. Because the components were standardized, a one-SD increase in authoritarianism increased the estimated benevolence slope by 0.400 units. Ambivalent psychological safety was positively associated with resource depletion (b = 0.390, SE = 0.047, *p* < 0.001, 95% CI [0.298, 0.482]), supporting H2. The ambivalent psychological safety x surface acting interaction was also significant (b = 0.151, SE = 0.046, *p* = 0.001, 95% CI [0.061, 0.241]); thus a one-SD increase in surface acting increased the estimated ambivalent-safety slope by 0.151 units, supporting H3. Resource depletion was negatively associated with promotive voice (b = −0.442, SE = 0.050, *p* < 0.001, 95% CI [−0.540, −0.344]) and prohibitive voice (b = −0.247, SE = 0.054, *p* < 0.001, 95% CI [−0.353, −0.141]), supporting the directional components of H4a and H4b.

Figure 4 decomposes the H1 interaction using organization-clustered inference. At low authoritarianism (−1 SD), the simple benevolence slope was negative (b = −0.240, SE = 0.066, *t*(17) = −3.61, *p* = 0.002, 95% CI [−0.379, −0.100]); at high authoritarianism (+1 SD), the slope was positive and substantially stronger (b = 0.561, SE = 0.085, *t*(17) = 6.59, *p* < 0.001, 95% CI [0.382, 0.741]). The predicted standardized value was highest in the high-benevolence/high-authoritarianism condition (0.851), directly illustrating the configuration proposed in H1.

**Figure 4:**
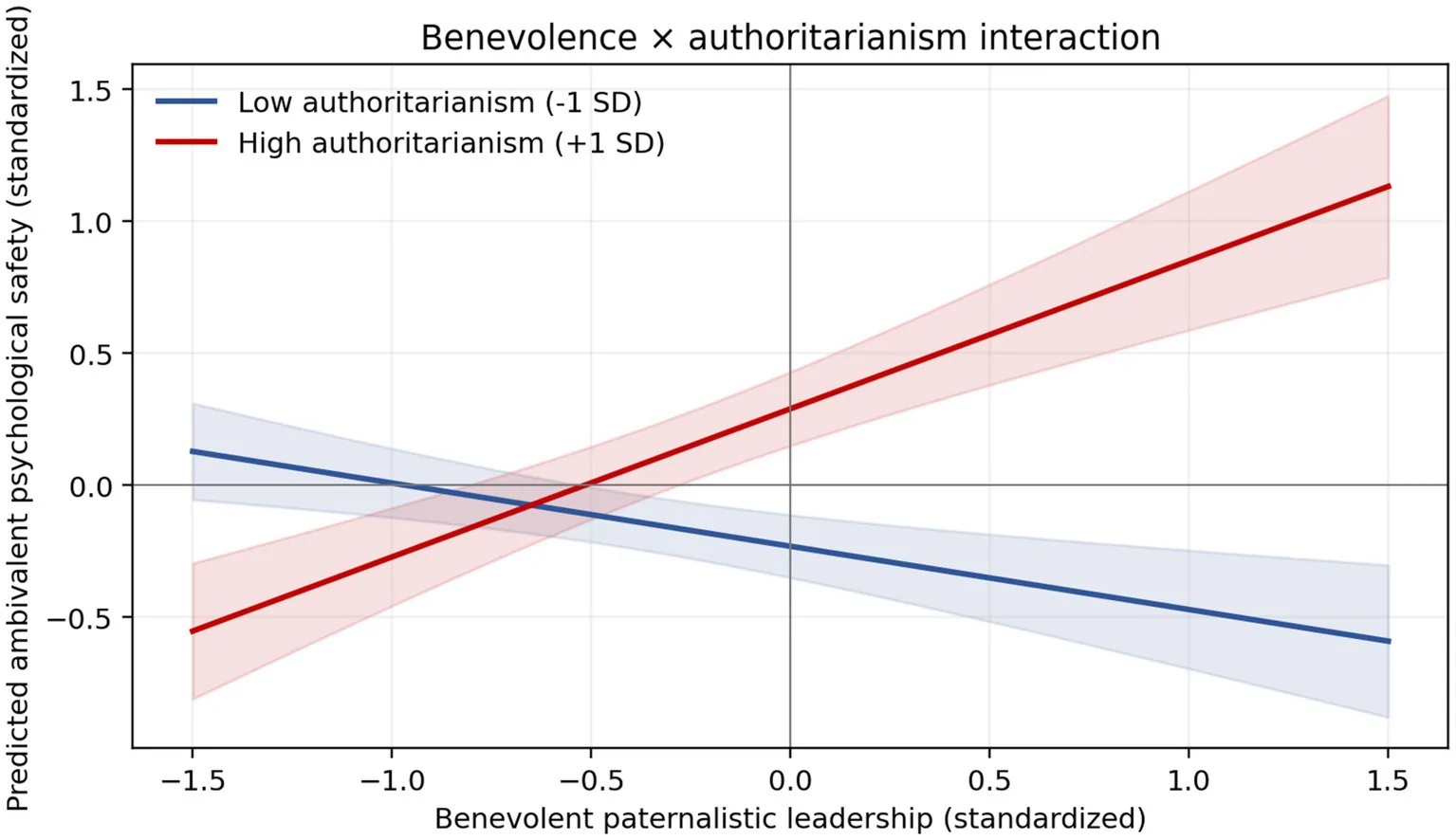
Simple slopes for the benevolence *x* authoritarianism interaction predicting ambivalent psychological safety. Shaded regions are 95% confidence bands based on organization-clustered covariance estimates.

The difference between the depletion-to-voice paths was tested directly in a stacked-outcome model rather than inferred from separate significance tests. With promotive voice as the reference outcome, the prohibitive-by-depletion interaction was positive (difference = 0.194), confirming that the prohibitive association was less negative. The contrast remained significant with supervisor-clustered inference (SE = 0.074, *t*(71) = 2.62, *p* = 0.011, 95% CI [0.046, 0.343]) and organization-clustered inference (SE = 0.064, *t*(17) = 3.06, *p* = 0.007, 95% CI [0.060, 0.329]). Thus, H4b’s predicted asymmetry is supported after accounting for non-independence.

The latent-product SEM robustness check produced the same inference pattern: the benevolence x authoritarianism association remained positive (b = 0.376, SE = 0.050, *p* < 0.001), ambivalent psychological safety remained positively associated with depletion (b = 0.370, SE = 0.053, *p* < 0.001), the ambivalent safety x surface acting interaction remained significant (b = 0.139, SE = 0.052, *p* = 0.007), and depletion remained negatively associated with promotive voice (b = −0.415, SE = 0.056, *p* < 0.001) and prohibitive voice (b = −0.232, SE = 0.060, *p* < 0.001).

As shown in Table 6 and Figure 5, the organization-cluster bootstrap estimates were consistent with H5. The indirect association with promotive voice was negative under low surface acting (−0.042, 95% CI [−0.079, −0.011]) and stronger under high surface acting (−0.096, 95% CI [−0.124, −0.070]). The corresponding association with prohibitive voice moved from −0.024 (95% CI [−0.042, −0.007]) to −0.054 (95% CI [−0.076, −0.029]). At high surface acting, the promotive-minus-prohibitive contrast was −0.042, 95% CI [−0.076, −0.018]. The indices of moderated mediation were also negative for promotive voice (−0.027, 95% CI [−0.043, −0.012]) and prohibitive voice (−0.015, 95% CI [−0.028, −0.005]).

**Table 6:** Conditional indirect effects.

Outcome	Surface acting level	Indirect effect	Boot SE	95% CI	Interpretation
Promotive voice	Low (−1 SD)	−0.042	0.017	[−0.079, −0.011]	Negative indirect association
Promotive voice	High (+1 SD)	−0.096	0.014	[−0.124, −0.070]	Stronger negative association
Prohibitive voice	Low (−1 SD)	−0.024	0.009	[−0.042, −0.007]	Negative indirect association
Prohibitive voice	High (+1 SD)	−0.054	0.012	[−0.076, −0.029]	Weaker than promotive voice
Difference	Promotive vs. prohibitive, high surface acting	−0.042	0.015	[−0.076, −0.018]	Promotive path more negative

**Figure 5:**
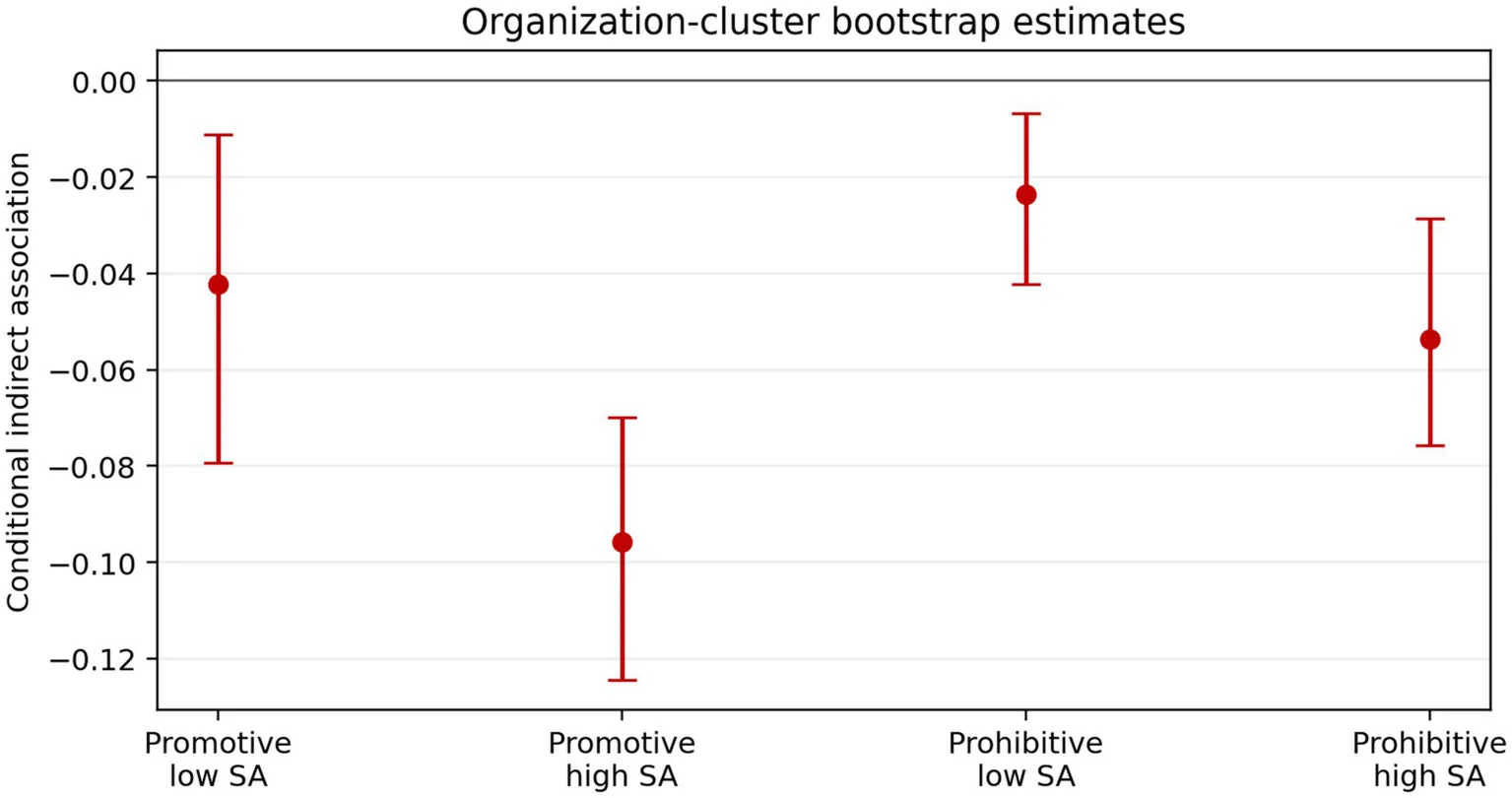
Conditional indirect associations with 95% percentile confidence intervals from 5,000 organization-cluster bootstrap samples. SA = surface acting. Resampling preserved organizations, supervisors, and employees; the estimates remain observational.

Substantively, the strongest negative indirect association involved promotive voice under high emotional-labor demand. This pattern remained present when entire organizations, including their supervisors and employees, were resampled. It is consistent with the idea that promotive voice requires discretionary creative energy, whereas prohibitive voice may sometimes be sustained by immediate risk perception. The pattern remains associative because cluster-aware inference addresses dependence, not causal identification.

### 4.4 Cluster-robustness and direct effect-contrast checks

Nested-ANOVA variance components indicated modest dependence. The estimated organization ICC was 0.000 for ambivalent psychological safety and depletion, 0.015 for promotive voice, and 0.000 for prohibitive voice. The additional supervisor component was 0.012 for ambivalent psychological safety and 0.064 for depletion and was estimated as zero for both voice outcomes. Because near-zero variance estimates can occur at the boundary, inference was nevertheless re-estimated at both clustering levels rather than assuming independence. Table 7 reports the cluster-robust results.

**Table 7:** Focal estimates with supervisor- and organization-clustered inference.

Effect	*b*	Supervisor SE	Supervisor p	Supervisor 95% CI	Organization SE	Organization *p*	Organization 95% CI
H1 interaction	0.400	0.046	<0.001	[0.309, 0.492]	0.046	<0.001	[0.304, 0.497]
H2 ambivalence	0.390	0.045	<0.001	[0.301, 0.479]	0.049	<0.001	[0.286, 0.494]
H3 moderation	0.151	0.045	0.001	[0.061, 0.242]	0.047	0.005	[0.052, 0.251]
H4a promotive	−0.442	0.050	<0.001	[−0.541, −0.342]	0.034	<0.001	[−0.514, −0.369]
H4b prohibitive	−0.247	0.054	<0.001	[−0.354, −0.140]	0.056	<0.001	[−0.366, −0.128]
H4 difference	0.194	0.074	0.011	[0.046, 0.343]	0.064	0.007	[0.060, 0.329]

CR1 finite-sample corrections were applied; *t*-tests use cluster-count minus one degrees of freedom. The H4 difference is prohibitive minus promotive, so a positive coefficient indicates that the prohibitive association is less negative. All focal paths and the H4 contrast retained their direction and statistical significance at both clustering levels.

## 5 Discussion

The results are consistent with the proposed care-control-resource model. The joint presence of benevolent care and authoritarian control was associated with higher ambivalent psychological safety; ambivalent safety was associated with greater emotional resource depletion, especially at higher surface acting; and depletion was more strongly negatively associated with promotive than prohibitive voice. These temporally ordered associations support the proposed mechanism but do not demonstrate that the leadership configuration caused subsequent depletion or voice behavior.

The findings help explain why benevolent leadership cues may fail to protect voice when they are paired with strong authoritarian cues. Benevolence can make employees feel seen, supported, and obligated, but authoritarianism can make them cautious about whether disagreement will be interpreted as insubordination. The combination need not average into a moderate leadership climate; the observed interaction is instead consistent with heightened ambivalence and the emotional work required to manage it.

This interpretation is especially important in service organizations undergoing AI transformation and wage-pressure adjustment. Leaders may believe that personal care is enough to soften stricter control, tighter scheduling, or more intensive monitoring. The findings suggest a more cautious conclusion: care may reduce some relational distance, but it does not automatically remove the resource costs of control when employees are deciding whether to challenge, suggest, or warn.

The results suggest that a leader’s benevolent concern does not automatically make control harmless. Employees may appreciate personal care while still conserving resources because they are unsure whether challenge is acceptable. This interpretation is especially relevant to service organizations attempting to introduce AI-enabled work redesign while asking employees to maintain customer-facing warmth, absorb wage-related productivity expectations, and continue offering improvement ideas.

The moderating role of surface acting clarifies the proposed resource-competition mechanism. Employees who regulate outward display while inner feelings remain unchanged must monitor discrepancies, inhibit spontaneous expression, and maintain a service-appropriate response. Ambivalent leader cues require a parallel cycle of scanning care and control signals, anticipating the leader’s likely reaction, and editing a potential voice message. The positive interaction is consistent with these simultaneous demands competing for self-regulatory capacity. Surface acting therefore changes the resource context in which leadership ambiguity is processed; it is not interpreted here as a stable trait or as proof of depletion causation.

The stronger negative path for promotive voice indicates that resource depletion may first reduce employees’ willingness to offer future-oriented improvement suggestions. Prohibitive voice was also negatively related to depletion, but the association was weaker, consistent with the idea that risk-focused warnings may remain partly activated by perceived threat or problem urgency. Thus, treating voice as a single construct would understate the theoretical importance of voice form.

This asymmetry has practical meaning. Many AI and service-redesign initiatives depend heavily on promotive voice because leaders need employees to suggest how new tools can be adjusted to real customer interactions. If the employees closest to the service process are depleted, organizations may lose the very suggestions needed to make transformation work. Prohibitive voice remains important for detecting risks, but a system that only preserves warnings while losing improvement ideas may become reactive rather than adaptive.

### 5.1 Theoretical implications

The study contributes to paternalistic leadership research by modeling benevolence and authoritarianism as a configuration. Prior work often evaluates whether benevolence has beneficial effects and authoritarianism has harmful effects. The present model shows why their combination deserves separate attention: benevolence may coexist with authoritarian control in ways that generate ambivalent rather than uniformly safe employee perceptions. This extends paternalistic leadership theory by specifying a mechanism through which care and control jointly shape employee behavior.

The study also contributes to psychological safety research. Psychological safety is commonly framed as a climate or perception that enables learning behavior. The present study argues that employees may experience safety ambivalently when leader cues are mixed. This expands the concept beyond a high-low continuum and suggests that the coexistence of safety and caution can itself be psychologically demanding. Ambivalent safety is therefore not merely a weaker form of safety; it is a qualitatively different state with resource implications.

A third contribution concerns employee voice. By comparing promotive and prohibitive voice, the study responds to calls to examine voice forms separately rather than assuming a single speaking-up tendency. The findings suggest that depletion has a stronger negative relationship with promotive voice, consistent with the idea that improvement suggestions require more discretionary cognitive and emotional energy than risk warnings. This provides a resource-based explanation for why some employees may continue to flag problems while withdrawing from constructive innovation-oriented suggestions.

Finally, the study contributes to understanding contemporary service work. AI transformation and minimum-wage pressure are often discussed as strategic or operational issues, but the findings highlight their interpersonal consequences. When transformation pressure encourages leaders to combine support with control, employees may experience voice as emotionally expensive. This places leadership behavior at the center of whether service organizations can learn from employees during periods of technological and labor-cost change.

### 5.2 Practical implications

#### 5.2.1 Supervisor-level actions

At the supervisor level, care should be paired with explicit behavioral permission to disagree. During AI-enabled redesign or productivity changes, supervisors can invite counterarguments, thank employees for warnings, explain how a suggestion will be evaluated, and separate respectful challenge from disobedience. These behaviors target the contradictory care-control cue that underlies ambivalent psychological safety.

#### 5.2.2 Organization-level actions

At the organization level, formal routines can make voice expectations less dependent on a single supervisor’s discretion. Structured debriefs after AI-tool implementation, anonymous early-warning channels, documented response protocols, and recurring improvement meetings can specify when and how challenge is expected. Recovery breaks, staffing practices, and realistic display rules address the separate resource burden associated with surface acting. Thus, voice-system interventions primarily reduce ambiguity, whereas recovery and emotional-labor interventions primarily protect depleted resources.

### 5.3 Limitations and future research

Several limitations qualify the findings. First, the design is observational. Temporal separation, supervisor-rated outcomes, cluster-robust inference, and organization-cluster bootstrap resampling reduce specific methodological concerns but do not establish causality, rule out time-varying confounding, or exclude reciprocal processes. Second, the organization-level analysis contains only 18 clusters. Finite-sample corrections and whole-organization resampling were used, and conclusions were unchanged, but higher-level variance estimates and cluster-based confidence intervals remain less precise than they would be with more organizations. Third, several mechanisms and controls remain employee-reported. Fourth, the service-sector sample limits generalization. Fifth, the six-item ambivalent psychological safety measure showed reliability and factorial distinctiveness, but fuller construct assessment should include convergent, discriminant, criterion, and incremental validity against conventional psychological safety, fear of speaking up, role conflict, and general affective ambivalence.

Cultural and institutional boundary conditions require particular attention. In Malaysia’s multiethnic service workforce, employees may differ in the legitimacy they grant hierarchy, the relational meaning they attach to leader care, and the indirectness they regard as appropriate when challenging authority. These possibilities should not be reduced to a single national-culture score. Institutionally, minimum-wage compliance, labor availability, customer-service display rules, union representation, and the transparency of AI-enabled monitoring may each alter whether authoritarian direction is interpreted as legitimate coordination or interpersonal threat. Comparative research across Malaysian regions, subsectors, ethnic and language groups, and countries should test measurement invariance before comparing structural paths. Team voice climate, role expertise, newcomer status, and labor-market alternatives are additional moderators that may amplify or buffer the care-control tension.

Another useful direction is to examine leader intentions and employee interpretations simultaneously and repeatedly. A supervisor may intend benevolence and control as complementary, while employees may interpret the same combination as uncertain and resource-demanding. Experience sampling could establish temporal precedence over shorter intervals; experiments could manipulate care and control cues; and qualitative process studies could show how employees resolve mixed signals in actual voice episodes. Multilevel designs with adequate numbers of supervisors and organizations could then distinguish employee-specific ambivalence from shared team or organizational climates.

## 6 Conclusion

This study provides evidence consistent with a resource account of paternalistic leaders’ care-control duality in Malaysian service work. Under AI transformation and minimum-wage pressure, the joint presence of benevolent and authoritarian cues was associated with ambivalent psychological safety; ambivalent safety was associated with emotional resource depletion, particularly when surface acting was high; and depletion was more strongly negatively associated with promotive than prohibitive voice. These associations and the voice-path difference remained significant with supervisor- and organization-clustered inference, and the moderated indirect pattern remained present in 5,000 organization-cluster bootstrap samples. The findings do not establish causality, but they indicate that the results are not an artifact of treating nested observations as independent.

## Data Availability

The raw data supporting the conclusions of this article will be made available by the authors, without undue reservation.
